# Supplementary material for: A Global Women’s Rugby Union Web-Based Survey
Source: Int J Environ Res Public Health. 2023 Apr 12;20(8):5475. doi: 10.3390/ijerph20085475 (PMC10138678; doi:10.3390/ijerph20085475)
Supplement: Supplementary file 1 [file ijerph-20-05475-s001.zip › ijerph-2161427-supplementary.pdf]

Swansea University

Women's Rugby Union Player Survey Questionnaire 2020

Page 1: PARTICIPANT INFORMATION SHEET - PLAYERS

PARTICIPANT INFORMATION SHEET - PLAYERS (Version 1.1, Date: 29 /06/2020)

Project Title: Building an Evidence Base for Women-Specific Training and Injury Identification in Rugby Union - Global Survey

Thank you for your time in taking part in the women's rugby union survey for 2020. The objective of this survey is to help to bridge the substantial gender data gap which exists in medical and sport science, particularly in contact sports. You are free to contact the research team at any time for any questions or ideas (details below):

Contact Details:

Dr Elisabeth Williams, ASTEM, College of Engineering, Bay Campus, Swansea University, Fabian Way, Swansea, SA18EN, United Kingdom: e.m.p.williams@swansea.ac.uk

Alternative Contacts: Dr Anna Stodter - anna.stodter@anglia.ac.uk; Dr Izzy Moore - imoore@cardiffmet.ac.uk; Dr Genevieve Williams - G.K.R.Williams@exeter.ac.uk

Invitation Paragraph

My name is Dr Elisabeth Williams and I am a senior lecturer in applied biomechanics at Swansea University in Wales, U.K. On behalf of the research team, we would like to thank you for your time and interest in completing this questionnaire.

What is the purpose of the study?

The aim of this survey questionnaire is to gather data about women rugby players who participate at all levels; local club, university, elite, professional and international. You will be aware that

professionalisation of women's rugby has come 33 years after the male game. Currently, worldwide women's participation in rugby is rising by 28% per year, with 2.7 million registered players at the end of 2018. As rugby has historically been a male-dominated sport, most research about training techniques and injury risk has focussed on male athletes.

Male-based findings in sports and medicine are routinely generalised to women, even when there is little to no evidence about how women respond. This has created a significant gender data gap, and training programs are a good example of this. What this ultimately means is 'women are not small men' and we need to ensure we are training women appropriately to make them better, safer athletes. This very much applies to sports related mild traumatic brain injury (mTBI), which is a serious problem in contact sports like rugby.

There are many physical differences between males and females, in addition to playing opportunities and access to expert coaching, which may compromise the safety and performance of women rugby players. Reports have shown women to be 2.6 times more likely to suffer a concussion in sport. Research shows that female athletes take longer to return to play, have worse symptoms and are more likely to report dizziness, fatigue and difficulty concentrating than males. Differences between male and female head/neck physiology, neck strength and neuronal structure in the brain have been linked to a greater head injury severity in females following rugby impacts.

Why have I been chosen?

You have been approached via women's rugby social media platforms to participate in this study as you are a women's rugby player, over the age of 18. We would like to know about your experiences, the anthropometrics of different playing positions, injury patterns and health monitoring practices in particular. The data you provide will help us to create an evidence base to develop targeted, women-specific interventions to improve player safety. We hope to have as many women rugby players as possible complete this survey in order to build a valuable and informative dataset that we can all benefit from.

Taking part in this study is entirely voluntary. The questionnaire responses are completely anonymous, even to the research team. **As your responses for each question are automatically saved when you answer them, and no identifiable information is recorded in this study, we are unable to withdraw your data once you have provided a response.**

What will happen to me if I take part?

Participation in this study involves completing the anonymous questionnaire which follows this document, after you click to confirm that you are over 18, fit the criteria for the study and agree to participate. The questionnaire is in three sections:

- **Section One:** Demographics, playing experience and positions, playing level and basic health

information.
- **Section Two:** Concussion history, strength and conditioning, injury prevention training and injury management.
- **Section Three:** Women-specific concussion symptoms, the effects of menstrual cycle on training, performance and injuries, female physiology, medical support and financial resources in women's rugby union.

The questionnaire should take you approximately 20 minutes for section 1 and depending on the length of your answers, 20-30 minutes each for sections 2 and 3. By answering all the sections of the questionnaire, you will help us to tackle the significant gender data gap.

What are the possible disadvantages of taking part?

Some questions will ask you about previous concussions or possible concussions you may have had, both while playing rugby and in other aspects of your life. This may trigger upsetting memories, depending on the circumstances surrounding your injuries. If you do feel upset by any content in the questionnaire, you are advised to contact someone you trust and you are always welcome to contact anyone from the research team, whose email addresses are provided above.

6. What are the possible benefits of taking part?

By taking part in this study, you will be making an invaluable contribution to the much-needed knowledge about women's rugby union. This information will be used to improve training strategies to minimise the risk of brain injury to women rugby players and other contact sport athletes. The data gathered from this questionnaire will also help us understand what most women rugby players know about how their bodies respond to trauma and to different training stimuli at different phases of the menstrual cycle. With that information we can work with rugby organisations to improve training practices and guidelines specific to women athletes. In addition to publishing our generalised findings in open-source journals, we will be making our generalised findings available on Twitter: @TBI-ResearchNet.

7**. Will my taking part in the study be kept confidential?**

Due to the nature of this anonymous survey, your identity will not be recorded. The anonymised data will be kept by the research team and used both directly in publishable studies and as pilot information for three years. After this date, the data will be stored for a further four years for reference for future work in this area. All anonymised data will be kept on a secure cloud platform, accessible only by the research team.

Here is a news article link from early 2020 to provide you with more context for this study:

https://www-bbc-co-uk.cdn.ampproject.org/c/s/www.bbc.co.uk/news/amp/uk-wales-51434749

Data Protection and Confidentiality: Your data will be processed in accordance with the Data Protection Act 2018 and the General Data Protection Regulation 2016 (GDPR). All information collected about you will be kept strictly confidential. Your data will only be viewed by the researcher/research team. All electronic data will be stored on a password-protected computer file at Swansea University. Your consent information does not require your name to eliminate risk in the event of a data breach. Please note that as data is being collected online, once the data has been submitted online you will be unable to withdraw your information. **Data Protection Privacy Notice:** The data controller for this project will be Swansea University. The University Data Protection Officer provides oversight of university activities involving the processing of personal data, and can be contacted at the Vice-Chancellors Office. Your personal data will be processed for the purposes outlined in this information sheet. Standard ethical procedures will involve you providing your consent to participate in this study by completing the consent form that has been provided to you. The legal basis that we will rely on to process your personal data will be necessary for the performance of a task carried out in the public interest. This public interest justification is approved by the College of Engineering Research Ethics Committee, Swansea University. The legal basis that we will rely on to process special categories of data will be processing is necessary for archiving purposes in the public interest, scientific or historical research purposes or statistical purposes. **How long will your information be held?** We will hold any personal data and special categories of data for seven years. **What are your rights?** Please visit the University Data Protection webpages for further information in relation to your rights. Any requests or objections should be made in writing to the University Data Protection Officer: University Compliance Officer (FOI/DP), Vice-Chancellor's Office, Swansea University, Singleton Park, Swansea, SA2 8PP. Email: dataprotection@swansea.ac.uk **How to make a complaint:** If you are unhappy with the way in which your personal data has been processed, you may in the first instance contact the University Data Protection Officer using the contact details above. If you remain dissatisfied, then you have the right to apply directly to the Information Commissioner for a decision. The Information Commissioner can be contacted at: Information Commissioner's Office, Wycliffe House, Water Lane, Wilmslow, Cheshire, SK9 5AF, www.ico.org.uk 8**. What if I have any questions?** Re-iterate that further information can be obtained from the researcher contact stated above. Also state that the project has been approved by the College of Engineering Research Ethics Committee at Swansea University. If you have any questions regarding this, any complaint, or concerns about the ethics of this research please contact Dr Andrew Bloodworth, Chair of the College of Engineering Research Ethics Committee, Swansea University. A.J.Bloodworth@swansea.ac.uk. The institutional contact for reporting cases of research conduct is Registrar & Chief Operating Officer Mr Andrew Rhodes. Email: researchmisconduct@swansea.ac.uk. Further details are available at the Swansea University webpages.

1. I confirm that I have read and understood the information sheet dated 29/06/2020, version number 1.1 and that I have the opportunity to contact members of the research team to ask questions.   I understand that my participation is voluntary, and the questionnaire responses are completely anonymous, even to the research team.   I understand that my responses for each question are automatically saved when I answer them, and no identifiable information is recorded in this study. I understand that I am unable to withdraw my data once I have provided a response. I understand that data obtained may be looked at by responsible individuals from Swansea University or from regulatory authorities where it is relevant to my taking part in research. I give permission for these individuals to have access to these records. I understand that data I provide may be used

in reports and academic publications in anonymous fashion.

- [ ] I agree to take part in the above study

Page 2: Section 1: Demographics and Rugby History

2. What country are you based in?

2.a. If you selected Other, please specify:

3. Please state the region you currently play for (optional)

4. What is your current age (years)?

5. What is your height?

6. What is your body weight?

7. At what age did you start playing rugby?

8. How many years have you been taking part in rugby matches?

9. Please select what best describes your playing level (select all that apply)

Please select at least 1 answer(s).

- [ ] Recreational
- [ ] University second division
- [ ] Club second division
- [ ] University first division
- [ ] Club first division
- [ ] Premier club
- [ ] National team
- [ ] Other

9.a. If you selected Other, please specify:

10. What are your current/most recent playing positions (select all that apply)

- [ ] 1
- [ ] 2
- [ ] 3
- [ ] 4
- [ ] 5
- [ ] 6
- [ ] 7
- [ ] 8
- [ ] 9
- [ ] 10
- [ ] 11
- [ ] 12
- [ ] 13
- [ ] 14
- [ ] 15

11. Do you play Rugby Sevens?

- ○ Yes
- ○ No

11.a. What are your current/most recent sevens playing positions (select all that apply)

[ ]

12. Do you **currently** compete in any other sport in addition to rugby?

- ○ Yes
- ○ No

12.a. Which other sports do you currently compete in?

- ☐ Football/Soccer
- ☐ Netball
- ☐ Swimming
- ☐ Athletics
- ☐ Field Hockey
- ☐ Martial Arts
- ☐ Tennis
- ☐ Cricket
- ☐ Gymnastics
- ☐ Other

12.b. How long have you been competing in this sport?

- ○ Less than one year
- ○ 1-3 years
- ○ 4-6 years
- ○ 6-10 years
- ○ 10+ years

12.c. What level do you compete at in your other sport(s)?

- ☐ Recreational
- ☐ University/Collegiate
- ☐ Club first division
- ☐ Premier club
- ☐ International

☐ Other

12.c.i. If you selected Other, please specify:

13. Prior to playing rugby, did you compete in any other sport(s)?

○ Yes
○ No

13.a. What sport(s) did you used to compete in? (select all that apply)

Please select at least 1 answer(s).

☐ Football /soccer
☐ Netball
☐ Swimming
☐ Athletics
☐ Field Hockey
☐ Martial Arts
☐ Cricket
☐ Tennis
☐ Badminton
☐ Water Sports
☐ Snow Sports
☐ Lacrosse
☐ Other

13.a.i. If you selected Other, please specify:

13.b. How long have you been competing in this/these sport(s)?

- ☐ Less than one year
- ☐ 1-3 years
- ☐ 4-6 years
- ☐ 6-10 years
- ☐ 10+ years

13.c. At what level did you compete in your other sport(s)?

Please select at least 1 answer(s).

- ☐ Recreational
- ☐ University
- ☐ Club first division
- ☐ Premier club
- ☐ National team / International
- ☐ Other

13.c.i. If you selected Other, please specify:

14. Are you familiar with the term concussion?

- ○ Yes
- ○ No

15. Which of the following are common signs of concussion? (select all that apply)

- ☐ Arm pain
- ☐ Chest pain
- ☐ Confusion
- ☐ Cut to the face
- ☐ Difficulty concentrating
- ☐ Dizziness
- ☐ Drowsiness
- ☐ Feeling or being sick
- ☐ Headache
- ☐ Knocked out
- ☐ Memory loss
- ☐ Neck pain
- ☐ Nosebleed
- ☐ Stomach cramps

16. Please select all statements you think are true/correct:

- ☐ Concussion is caused by an impact to the head
- ☐ Concussion is caused by an impact to the body
- ☐ Concussion only happens when a person is knocked out/loses consciousness
- ☐ Concussion only happens in rugby/contact sport
- ☐ Concussion can be caused by a direct or indirect blow to the head
- ☐ Most concussions don’t involve loss of consciousness

- [ ] All concussions get reported and documented
- [ ] Most concussions go unreported and undiagnosed
- [ ] Other

16.a. Further comments:

17. Have you received any concussion education through your rugby team? *(select all that apply to you).* ✱ *Required*

- [ ] None, it's never been mentioned
- [ ] My coach has talked to me about it on one or two occasions
- [ ] A member of the medical team has talked to me about it on one or two occasions
- [ ] I have had a concussion seminar in the past
- [ ] I have taken part in concussion seminars every season
- [ ] I have completed an online concussion education program every season (such as England Rugby's 'Headcase' or World Rugby Concussion Management)
- [ ] Other

17.a. If you selected Other, please specify:

18. Have you ever had a concussion **while playing rugby**?

- ○ Yes

- No
- I don't know

18.a. How did the concussion(s) happen? (select all that apply, for example: I was the tackler AND hit my head on the ground)

- I was tackled
- I was the tackler
- I was in a ruck
- My head collided with another player’s head
- My head collided with another player’s shoulder bone/hip bone/knee bone/foot)
- My head collided with another player’s soft body part (.g. stomach)
- My head collided with the ground
- I don’t know
- Other

18.a.i. If you selected Other, please specify:

18.b. At what age did you experience this/these concussion(s)? (select all that apply)

- Under 10 years
- 11 - 13 years
- 14 -16 years
- 17 - 19 years
- 20 - 24 years
- 25 - 29 years
- 30 - 34 years
- 35 - 39 years

- [ ] 40 - 44 yerars
- [ ] 45 - 49 years
- [ ] 50+ years

18.c. Was/were your concussion(s) diagnosed by a medical professional?

- [ ] Yes
- [ ] No
- [ ] Other

18.c.i. If you selected Other, please specify:

____

18.c.ii. Was your concussion(s) reported to a medical professional?

- ( ) Yes
- ( ) No

18.c.ii.a. Why was your concussion not reported to a medical professional?

- [ ] I chose not to report it
- [ ] I did not realise at the time
- [ ] I didn’t think it needed medical attention, it would just get better with rest
- [ ] Other

18.c.ii.a.i. If you selected Other, please specify:

____

19. Have you ever had a concussion **while taking part in other sports?**

- Yes
- No

19.a. In which sport(s) did this/these concussion(s) happen and how did it happen?

19.b. At what age(s) did you experience this/these concussion(s)?

- Under 10 years
- 11-13 years
- 14 - 16 years
- 17 - 19 years
- 20 - 24 years
- 25 - 29 years
- 30 - 34 years
- 35 - 39 years
- 40 - 44 years
- 45 - 49 years
- 50 + years

19.c. Was/were your concussion(s) diagnosed by a medical professional?

- Yes
- No

19.c.i. Was/were your concussion(s) reported to a medical professional?

- Yes
- No

19.c.i.a. Why was/were your concussion(s) not reported to a medical professional?

- I chose not to report it
- I did not realise at the time
- I didn’t think it needed medical attention, it would just get better with rest
- Other

19.c.i.a.i. If you selected Other, please specify:

20. Have you ever / do you think you have ever had a concussion **while taking part in other activities or any aspect of your life?**

- Yes
- No

20.a. Under what circumstances or taking part in what activity did this occur (e.g fall from bike/horse, altercation, car accident)?

20.b. At what age(s) did you experience this/these concussion(s)?

- Under 10 years

- [ ] 11-13 years
- [ ] 14 - 16 years
- [ ] 17 - 19 years
- [ ] 20 - 24 years
- [ ] 25 - 29 years
- [ ] 30 - 34 years
- [ ] 35 - 39 years
- [ ] 40 - 44 years
- [ ] 45 - 49 years
- [ ] 50 + years

20.c. Was it diagnosed by a medical professional?

- ○ Yes
- ○ No

20.c.i. Was/were your concussion(s) reported to a medical professional?

- ○ Yes
- ○ No

20.c.i.a. Why was/were your concussion(s) not reported to a medical professional?

- [ ] I chose not to report it
- [ ] I did not realise at the time
- [ ] I didn’t think it needed medical attention, it would just get better with rest
- [ ] Other

20.c.i.a.i. If you selected Other, please specify:

21. How many concussions do you *think* you have had during your rugby career?

- 0
- 1
- 2
- 3
- 4
- 5
- 6
- 7
- 8
- 9
- 10+

Page 3: Section 2: Strength & Conditioning, Concussion History and Injury Management

22. When you first started playing rugby: Do you feel you were given sufficient contact training before your first match to ensure safe and effective contact?

- Yes
- No
- Other

22.a. Do you have any further comments on this?

23. When you first started playing rugby: Before your first match, approximately how many hours of full contact training were you given in total?

More info

- None
- One session of contact/tackle training
- Two sessions of contact/tackle training
- Three to five sessions of contact/tackle training
- Approximately one month of regular contact/tackle training
- Approximately two months of regular contact/tackle training
- More than two months of regular contact/tackle training
- Other

23.a. Do you have any further comments on this?

24. When you first started playing rugby: How confident did you feel with regard to contact technique and contact intensity going into your first match?

24.a. Do you have any further comment on this?

25. When you started playing rugby, did you complete any training on how to fall or land on the ground?

- Yes
- No
- Other

25.a. Do you have any further comments on this?

25.b. Has this falling technique training been completed again since you first started playing?

- ☐ No
- ☐ Yes, once per season
- ☐ Yes, once per month
- ☐ Yes, weekly
- ☐ Other

25.b.i. Do you have any further comments on this?

25.c. What was the fall techniques recommended (parachute, land on front, side etc), if you are unsure of the terms, please give a brief description:

26. In general, how long is your pre-season training block for your regular team? (from the time the players meet as a group)

- ☐ Training is only completed within the season
- ☐ One to two weeks (training less than 3 times per week)
- ☐ Three to six weeks (training less than 3 times per week)
- ☐ One to two weeks (training 3+ days per week)
- ☐ Three to four weeks
- ☐ Five to six weeks
- ☐ Other

26.a. Do you have any further comments on this?

27. What does your pre-season training typically include? (select all that apply)

- [ ] Running endurance and speed
- [ ] Strength and gym work
- [ ] Ball/passing skills
- [ ] Team tactics
- [ ] Contact drills and/or tackle technique
- [ ] Falling technique and/or body awareness training
- [ ] Active recovery sessions
- [ ] Other

27.a. Do you have any further comments on this?

28. During the pre-season, how many days rest from training do you normally have?

- ○ None
- ○ 1
- ○ 2
- ○ 3

- Other

28.a. It depends (please comment):

29. How many hours of contact activities (tackling, tackle technique, rucking etc.) do you do each week during the playing season for your regular team?

- None
- Less than one hour per week
- One dedicated contact session per week
- Two dedicated contact sessions per week
- Three dedicated contact sessions per week
- Four + dedicated contact sessions per week
- Other

29.a. Do you have any further comment on this?

30. How many hours of non-contact training (team fitness, passing, skills, agility drills, touch rugby) do you do each week during the playing season for your regular team?

- None
- Less than one hour during the week
- One dedicated non-contact session per week

- [ ] Two dedicated non-contact sessions per week
- [ ] Three dedicated non-contact sessions per week
- [ ] Four + dedicated non-contact sessions per week
- [ ] Other

30.a. Do you have any further comments on this?

31. Approximately how many hours of strength training (weights, plyometrics etc.) do you do each week during the playing season with your regular team?

- ○ None
- ○ Less than one hour during the week
- ○ One dedicated non-contact session per week
- ○ Two dedicated non-contact sessions per week
- ○ Three dedicated non-contact sessions per week
- ○ Four + dedicated non-contact sessions per week
- ○ Other

31.a. If you selected Other, please specify:

32. Alongside your rugby training how many hours per week do you spend doing your own

physical training during a typical week during the rugby season?

- ☐ None
- ☐ Up to one hour
- ☐ Up to two hours
- ☐ Up to three hours
- ☐ Up to four hours
- ☐ Up to five hours
- ☐ Up to six hours
- ☐ Up to seven hours
- ☐ Up to eight hours
- ☐ Up to nine hours
- ☐ Up to ten hours
- ☐ More than 10 hours
- ☐ Other

32.a. If you selected Other, please specify:

32.b. Of these hours you spend doing your own additional training, approximately how many hours to you spend doing strength/weight training?

32.c. Of these hours you spend doing your own additional training, approximately how many hours to you spend doing endurance/aerobic training?

32.d. Of these hours you spend doing your own additional training, approximately how many hours to you spend doing speed/sprint training?

32.e. Of these hours you spend doing your own additional training, approximately how many hours to you spend doing agility/coordination training?

33. Do you regularly use any recovery strategies after training/games? (select all that apply)

- ☐ None
- ☐ Yoga
- ☐ Hot/cold water immersion
- ☐ Leg compression
- ☐ Sports massage
- ☐ Special diets
- ☐ Low intensity exercise
- ☐ Stretching sessions
- ☐ Other

33.a. If you selected Other, please specify:

34. Typically (in a normal year) how many hours per week during your **off-season** do

you spend doing your own physical training?

34.a. Of the time you spend doing your own physical training during a typical off-season, how many hours per week would be **strength/weight training?**

34.b. Of the time you spend doing your own physical training during a typical off-season, how many hours per week would be **endurance/aerobic training?**

34.c. Of the time you spend doing your own physical training during a typical off-season, how many hours per week would be **speed/sprint training?**

34.d. Of the time you spend doing your own physical training during a typical off-season, how many hours per week would be **agility/coordination training?**

34.e. Of the time you spend doing your own physical training during a typical off-season, how many hours per week would be **contact/tackle training?**

35. How many hours of sleep do you usually get each night?

- Less than 6
- 6
- 7
- 8
- 9
- 9+

36. Do you regularly sleep / have a nap during the day?

- Yes
- No
- Other

36.a. If you selected Other, please specify:

37. Do you have a regular sleep/wake time?

- Yes
- No
- Other

37.a. If you selected Other, please specify:

38. How long do you have off all training at the end of the rugby season?

- <1 week
- 1-2 weeks
- 3-4 weeks
- 5-6 weeks
- Other

38.a. If you selected Other, please specify:

39. During your rugby career, have you received any education about the role of neck strength in minimising concussion injuries?

- Yes
- No
- Other

39.a. Do you have any further comments on this?

39.b. Have you ever done training focused on improving neck strength?

- No and I’m not interested in doing it
- No, but it's something I would be keen to try in the future
- Yes, our coaches encourage us to do basic neck strength exercises

- Yes, I do bodyweight neck strengthening exercises
- Yes, I do weighted neck strengthening exercises
- Other

39.b.i. Do you have any further comments on this?

40. During your rugby career, please list the injuries you have sustained and the number of times you have suffered each injury (e.g. torn hamstring *2, sprained ankle * etc.)

41. Following a previous rugby bodily injury (excluding possible concussion), did you feel you received adequate pitch-side medical provision in terms of injury identification?

- Yes
- No
- Not applicable
- Other

41.a. If you selected Other, please specify:

41.b. Following a previous rugby bodily injury (excluding possible concussion), did you feel you received adequate pitch-side medical provision in terms of injury management?

- ◯ Yes
- ◯ No
- ◯ Not applicable

41.b.i. Were you referred for further clinical assessment? (type yes or no)

42. When you return to play after a **bodily (non-head)** injury, are you confident that you are fit enough to play:

- ☐ Yes - I feel fully recovered
- ☐ Yes - I followed a full return-to-play protocol guided by a medical team
- ☐ Yes - I don't feel quite right but I think my injury will survive an upcoming match
- ☐ No - but I tried to play through it
- ☐ Other

42.a. If you selected Other, please specify:

43. Following a previous rugby **head injury or concussive** injury, did you feel you received adequate medical provision in terms of injury **identification:**

- Yes
- No
- Not applicable

43.a. Following a previous rugby **head injury or concussive injury**, did you feel you received adequate medical provision in terms of injury **management:**

- Yes
- No
- Not applicable
- Other

43.a.i. Do you have any further comments on this?

43.b. Following a previous rugby **head injury or concussive injury**, did you feel you received adequate medical provision in terms of injury **rehabilitation:**

- Yes
- No
- Not applicable
- Other

43.b.i. Do you have any further comments on this?

44. When you return to play rugby after a head injury or concussion, are you confident that you are fit enough to play?

- Yes - I feel fully recovered
- Yes - I followed a full return-to-play protocol guided by a medical team
- Yes - I don't feel quite right but I think my injury will survive an upcoming match
- No - but I tried to play through it
- Not applicable
- Other

44.a. Do you have any further comments on this?

45. Who develops/designs, promotes & manages your return to play (RTP) following injuries?

- Team doctor
- Team physio
- My own doctor (separate to team)
- My own physio (separate to team)
- Coaches - we just have a conversation
- Teammates - encouragement to come back
- I manage it myself, mostly based on how I feel and I decide
- Not applicable
- Other

45.a. If you selected Other, please specify:

46. If you were concussed during a game, would you be able to recognise it?

- Yes
- No
- Don't know
- Other

46.a. If you selected Other, please specify:

47. If you suspected that you suffered a concussion during a rugby match in the past, did you:

- Continue playing as normal
- Continue playing but trying to stay out of heavy contact
- Remove yourself from play
- Get removed from play by a coach/medic/team mate
- I’ve never suspected I had a concussion during a match

48. If you suspected your teammate had a concussion in a rugby game, would you report it?

- Yes
- No
- I don't know
- Other

48.a. If you selected Other, please specify:

49. When you play matches with your regular team, is there a medical professional present to identify suspected concussions?

- Yes
- No
- I don't know
- Other

49.a. If you selected Other, please specify:

50. If you have suffered a previous concussion, how long do you think it took for you to fully recover (feeling as well as you did before the injury, with no concussion)?

- Within 1 week

- Within 2 weeks
- Within 1 month
- Up to 3 months
- Beyond 3 months
- I am not the same as I was before the concussion/I have not fully recovered
- Not Applicable

50.a. If you selected Other, please specify:

51. Following a concussion have you felt pressure from your teammates to return to play?

- Yes - it probably shortened the time spent out of training/games
- Yes - but it did not affect the time it took me to return to play
- No, I did not feel pressure from anyone to return to play
- Other

51.a. Do you have any further comments on this?

52. Following a concussion have you felt pressure from your coaches to return to play?

- Yes - it probably shortened the time spent out of training/games

- Yes - but it did not affect the time it took me to return to play
- No, I did not feel pressure from anyone to return to play
- Other

52.a. If you selected Other, please specify:

53. When you have been concussed (or you or someone else thinks you've been concussed), do you put yourself under pressure to return to play?

- Yes
- No
- Not applicable

53.a. If you selected Other, please specify:

53.b. What is the top reason for returning to play?

- I don't like to miss games
- I miss the social network
- Without me my team will not win
- Other
- Not Applicable

53.b.i. If you selected Other, please specify:

54. Have you ever felt tired, irritable, confused, had concentration problems or other concussion symptoms following a match?

- Yes
- No
- Not applicable
- Other

54.a. If you selected Other, please specify:

54.b. Did you inform your coach?

- Yes, they brushed off my symptoms, encouraged me to continue playing/training
- Yes, they allowed me to decide on whether I continued playing/training
- Yes, they removed me from game/training and suggested I seek medical advice
- Yes, they actively helped me seek medical advice
- No, I didn't feel I could inform my coach

54.b.i. If you selected Other, please specify:

55. If you have previously experienced concussion, were you asked by a doctor/coach/medic what day of your menstrual cycle you were on?

- Yes
- No
- Not applicable
- Other

55.a. If you selected Other, please specify:

56. In your experience, do you think that the phase of your menstrual cycle can affect the severity of concussion symptoms and your subsequent recovery?

- Yes
- No
- I don't know
- Other

56.a. If you selected Other, please specify:

57. Are you aware of concussion return to play (RTP) guidelines?

- Yes
- No

57.a. What is the minimum amount of time before you can return to play following a concussion?

- I think I can return to play straight away
- At least 1 week
- At least 10 days
- At least 2 weeks
- At least 3 weeks
- At least 4 weeks
- Other

57.a.i. If you selected Other, please specify:

58. In your opinion, what do you think is the most common mechanism of head impact in women's rugby at the level you play at? *Examples can include direct contact with another player's head, direct contact between the head and the ground, direct contact as a tackler, as a ball carrier, head to knee, or foot of another player etc.*

Page 4: Section 3: Health and Wellbeing

59. Were you assigned the biological sex of female at birth'?

- Yes
- No

59.a. If no, are you currently undergoing hormonal treatments?

- Yes
- No

59.a.i. Please provide further details – please provide details of timeframes and dosages and any further details you believe to be relevant

59.b. Have you ever competed in sport in a male category?

- Yes
- No

59.c. Do you use hormonal contraception?

- Yes
- No

59.c.i. What form of hormonal contraceptive do you use?

- Combined oral

- Progesterone only (Mini Pill)
- Contraceptive patch
- Contraceptive injection
- Contraceptive implant
- Intrauterine device (IUD)
- Other

59.c.i.a. If you selected Other, please specify:

59.c.ii. How long have you used hormonal contraceptives for?

- <1 year
- 1-3 years
- 3-5 years
- 5 years +

59.c.iii. Do you experience bleeding? (referred to as withdrawal bleeds)

- Yes
- No
- Other

59.c.iii.a. If you selected Other, please specify:

59.c.iii.b. Do you continue to train and play during your withdrawal bleed?

- Yes

- No
- Other

59.c.iii.b.i. If you selected Other, please specify:

____

59.c.iii.c. Do you take medication to manage symptoms caused by your withdrawal bleed during training/games?

- Yes
- No

59.c.iii.c.i. Which medications do you take to manage these symptoms?

- Paracetamol
- Co Codamol
- Naproxen
- Ibuprofen
- Other

59.c.iii.c.i.a. If you selected Other, please specify:

____

59.c.iv. Do you track your reproductive cycle, which of the following applies to you?

- I track my cycle and relate this to my training program
- I track my cycle but don't relate it to my training
- I don’t track my cycle
- Other

59.c.iv.a. If you selected Other, please specify:

59.c.v. What symptoms of the reproductive cycle do you feel affect your training and performance? (please state below)

59.c.vi. Do you feel that your performance is altered when playing due to symptoms related to taking contraceptives?

- Yes
- No
- Other

59.c.vi.a. If you selected Other, please specify:

59.c.vi.b. Why do you feel that your performance is altered when playing due to symptoms related to taking contraceptives? (state below)

59.c.vii. Do you use hormonal contraceptives to be able to control or stop your periods

due to playing rugby?

- Yes
- No
- Other

59.c.vii.a. If you selected Other, please specify:

59.c.viii. Do you have a regular menstrual cycle?

- Yes
- No
- Other

59.c.viii.a. If you selected Other, please specify:

59.c.ix. How often do you have a period?

- 15-20 days
- 21-35 days
- 35 - 60 days
- Once or twice per year
- Less than once a year
- Not for the previous two years

59.c.x. Is the duration between your periods always the same length of time (±7days)?

- Yes
- Sometimes
- Rarely
- Never

59.c.xi. Do you continue to train/compete during your period?

- Yes
- No
- Other

59.c.xi.a. If you selected Other, please specify:

59.c.xii. Do you track your menstrual cycle, which of the following applies to you?

- I track my cycle and relate this to my training program
- I track my cycle but don't relate it to my training
- I don’t track my cycle

59.c.xiii. Do you take medication to manage symptoms caused by your period during training/games?

- Yes
- No
- Other

59.c.xiii.a. If you selected Other, please specify:

59.c.xiii.b. If Yes, which medications do you take? (select all that apply)

- Paracetamol
- Co Codamol
- Naproxen
- Ibuprofen
- Other

59.c.xiii.b.i. If you selected Other, please specify:

59.c.xiv. Do you feel that your training/performance is **enhanced** at certain times during your menstrual cycle/period?

- Yes
- No
- I don't know

59.c.xiv.a. When do you feel your performance is enhanced?

- In the week before your period
- During your period (when bleeding)
- The week following your period
- Other

59.c.xiv.a.i. If you selected Other, please specify:

59.c.xv. Do you feel that your training/performance is **impaired** at certain times during your menstrual cycle/period?

- Yes
- No
- I don't know

59.c.xv.a. When do you feel your performance is **impaired**?

- In the week before your period
- During your period (when bleeding)
- The week following your period
- Other

59.c.xv.a.i. If you selected Other, please specify:

59.c.xvi. What symptoms of the menstrual cycle do you feel affect your training and performance? (please state)

59.c.xvii. Have you experienced irregular or missed periods for more than 3 months?

- Yes
- No
- Other

59.c.xvii.a. If you selected Other, please specify:

59.c.xvii.b. If Yes, did this occur after experiencing concussion?

- Yes
- No
- Other

59.c.xvii.b.i. If you selected Other, please specify:

60. Provide any further information you feel is relevant to training, performance and injuries in relation to your menstrual cycle:

61. Do you receive pre-season health and/or medical screening,cognitive testing/screening or baseline concussion testing e.g. SCAT5?

- ○ Yes
- ○ No

61.a. If yes what did this include? (select all which apply)

- ☐ Baseline concussion testing
- ☐ Menstrual cycle/hormonal contraceptive history
- ☐ Fitness testing
- ☐ Injury history
- ☐ Other

61.a.i. If you selected Other, please specify:

62. Do you have any further comments to make about female rugby union training which you believe are important for future research?

Page 5: Thank you!

The research team would like to sincerely thank you for your time and participation in this world wide women's rugby union survey.

We believe that women athletes should be trained like women athletes, and not like they are just small men.

Please share with all the women rugby players you know, from anywhere, at any level!

Keep an eye out for our findings over the next six months by following @TBI_ResearchNet on Twitter.

Key for selection options

2 - What country are you based in?

- Algeria
- American Samoa
- Andorra
- Antigua and Barbuda
- Argentina
- Aruba
- Australia
- Austria
- Azerbaijan
- Bahamas
- Barbados
- Belarus
- Belgium
- Bermuda
- Bosnia and Herzegovina
- Botswana
- Brazil
- British Virgin Islands
- Brunei
- Bulgaria
- Burundi
- Cameroon

Canada
Cayman Islands
Chile
China
Chinese Taipei
Colombia
Cook Islands
Costa Rica
Croatia
Cuba
Curaçao
Cyprus
Czech Republic
Denmark
Dominican Republic
Ecuador
El Salvador
England
Estonia
Eswatini
Fiji
Finland
France
Georgia
Ghana
Germany
Greece
Guadeloupe
Guam
Guatemala
Guyana
Hong Kong
Hungary
Iceland
India
Indonesia
Iran
Ireland (Republic of)
Israel
Italy

Iceland
Ivory Coast
Jamaica
Japan
Kazakhstan
Kenya
Korea
Kyrgyzstan
Lao
Latvia
Liechtenstein
Lithuania
Luxembourg
Madagascar
Malaysia
Mali
Malta
Martique
Mauritius
Mexico
Moldova
Monaco
Mongolia
Montenegro
Morocco
Namibia
Netherlands
New Zealand
Nigeria
Niue
Northern Ireland
Norway
Pakistan
Panama
Papua New Guinea
Paraguay
Peru
Philippines
Poland
Portugal

Romania
Russia
Rwanda
Samoa
San Marino
Scotland
Sengal
Serbia
Singapore
Slovakia
Slovenia
Solomon Islands
South Africa
South
Spain
Sri Lanka
St. Kitts and Nevis
St. Lucia
St. Vincent & The Grenadines
Sweden
Switzerland
Tahiti
Tanzania
Thailand
Tonga
Trinidad and Tobago
Turkey
Turks and Caicos Islands
Ukraine
USA
United
Uruguay
Uzbekistan
Vanuata
Wales
Other

4 - What is your current age (years)?
18
19

20
21
22
23
24
25
26
27
28
29
30
31
32
33
34
35
36
37
38
39
40
41
42
43
44
45
46
47
48
49
50
51
52
53
54
55+

5 - What is your height?

140 cm / 4 ft, 7.1 in or below

141 cm / 4 ft, 7.5 in
142 cm / 4 ft, 7.9 in
143 cm / 4 ft, 8.3 in
144 cm / 4 ft, 8.7 in
145 cm / 4 ft, 9.1 in
146 cm / 4 ft, 9.5 in
147 cm / 4 ft, 9.9 in
148 cm / 4 ft, 10.3 in
149 cm / 4 ft, 10.7 in
150 cm / 4 ft, 11.1 in
151 cm / 4 ft, 11.4 in
152 cm / 4 ft, 11.8 in
153 cm / 5 ft, 0.2 in
154 cm / 5 ft, 0.6 in
155 cm / 5 ft, 1 in
156 cm / 5 ft, 1.4 in
157 cm / 5 ft, 1.8 in
158 cm / 5 ft, 2.2 in
159 cm / 5 ft, 2.6 in
160 cm / 5 ft, 3 in
161 cm / 5 ft, 3.4 in
162 cm / 5 ft, 3.8 in
163 cm / 5 ft, 4.2 in
164 cm / 5 ft, 4.6 in
165 cm / 5 ft, 5 in
166 cm / 5 ft, 5.4 in
167 cm / 5 ft, 5.7 in
168 cm / 5 ft, 6.1 in
169 cm / 5 ft, 6.5 in
170 cm / 5 ft, 6.9 in
171 cm / 5 ft, 7.3 in
172 cm / 5 ft, 7.7 in
173 cm / 5 ft, 8.1 in
174 cm / 5 ft, 8.5 in
175 cm / 5 ft, 8.9 in
176 cm / 5 ft, 9.3 in
177 cm / 5 ft, 9.7 in
178 cm / 5 ft, 10.1 in
179 cm / 5 ft, 10.5 in
180 cm / 5 ft, 10.9 in

- 181 cm / 5 ft, 11.3 in
- 182 cm / 5 ft, 11.7 in
- 183 cm / 6 ft, 0 in
- 184 cm / 6 ft, 0.4 in
- 185 cm / 6 ft, 0.8 in
- 186 cm / 6 ft, 1.2 in
- 187 cm / 6 ft, 1.6 in
- 188 cm / 6 ft, 2 in
- 189 cm / 6 ft, 2.4 in
- 190 cm / 6 ft, 2.8 in
- 191 cm / 6 ft, 3.2 in
- 192 cm / 6 ft, 3.6 in
- 193 cm / 6 ft, 4 in
- 194 cm / 6 ft, 4.4 in
- 195 cm / 6 ft, 4.8 in
- 196 cm / 6 ft, 5.2 in
- 197 cm / 6 ft, 5.6 in
- 198 cm / 6 ft, 6 in
- 199 cm / 6 ft, 6.3 in
- 200 cm / 6 ft, 6.7 in
- 201 cm / 6 ft, 7.1 in or above

6 - What is your body weight?

- < 40 kg / 88.2 lb
- 40 kg / 88.2 lb
- 41 kg / 90.4 lb
- 42 kg / 92.6 lb
- 43 kg / 94.8 lb
- 44 kg / 97.0 lb
- 45 kg / 99.2 lb
- 46 kg / 101.4 lb
- 47 kg / 103.6 lb
- 48 kg / 105.8 lb
- 49 kg / 108.0 lb
- 50 kg / 110.2 lb
- 51 kg / 112.4 lb
- 52 kg / 114.6 lb
- 53 kg / 116.8 lb
- 54 kg / 119.0 lb
- 55 kg / 121.3 lb

56 kg / 123.5 lb
57 kg / 125.7 lb
58 kg / 127.9 lb
59 kg / 130.1 lb
60 kg / 132.3 lb
61 kg / 134.5 lb
62 kg / 136.7 lb
63 kg / 138.9 lb
64 kg / 141.1 lb
65 kg / 143.3 lb
66 kg / 145.5 lb
67 kg / 147.7 lb
68 kg / 149.9 lb
69 kg / 152.1 lb
70 kg / 154.3 lb
71 kg / 156.5 lb
72 kg / 158.7 lb
73 kg / 160.9 lb
74 kg / 163.1 lb
75 kg / 165.3 lb
76 kg / 167.6 lb
77 kg / 169.8 lb
78 kg / 172.0 lb
79 kg / 174.2 lb
80 kg / 176.4 lb
81 kg / 178.6 lb
82 kg / 180.8 lb
83 kg / 183.0 lb
84 kg / 185.2 lb
85 kg / 187.4 lb
86 kg / 189.6 lb
87 kg / 191.8 lb
88 kg / 194.0 lb
89 kg / 196.2 lb
90 kg / 198.4 lb
91 kg / 200.6 lb
92 kg / 202.8 lb
93 kg / 205.0 lb
94 kg / 207.2 lb
95 kg / 209.4 lb

96 kg / 211.6 lb
97 kg / 213.8 lb
98 kg / 216.1 lb
99 kg / 218.3 lb
100 kg / 220.5 lb
101 kg / 22.7 lb
102 kg / 224.9 lb
103 kg / 227.1 lb
104 kg / 229.3 lb
105 kg / 231.5 lb
106 kg / 233.7 lb
107 kg / 235.9 lb
108 kg / 238.1 lb
109 kg / 240.3 lb
110 kg / 242.5 lb
111 kg / 244.7 lb
112 kg / 246.9 lb
113 kg / 249.1 lb
114 kg / 251.3 lb
115 kg / 253.5 lb
116 kg / 255.7 lb
117 kg / 257.9 lb
118 kg / 260.1 lb
119 kg / 262.4 lb
120 kg / 264.6 lb
121-125 kg / 266-276 lb
126-130kg / 277-287 lb
>130 kg / 287 lb

7 - At what age did you start playing rugby?

5
6
7
8
9
10
11
12
13
14

15
16
17
18
19
20
21
22
23
24
25
26
27
28
29
30
31
32
33
34
35
>35

8 - How many years have you been taking part in rugby matches?

<1
1
2
3
4
5
6
7
8
9
10
11
12
13
14

15
16
17
18
19
20
>20

11.a - What are your current/most recent sevens playing positions (select all that apply)

1
2
3
4
5
6
7

24 - When you first started playing rugby: How confident did you feel with regard to contact technique and contact intensity going into your first match?

Not confident at all (0/5)
A little confident (1/5)
Somewhat confident (2/5)
Relatively confident (3/5)
Comfortably confident (4/5)
Very confident (5/5)
Other

Swansea University

Women's Rugby Union Coach Survey Questionnaire 2020 English

Page 1: PARTICIPANT INFORMATION SHEET - COACHES

PARTICIPANT INFORMATION SHEET - COACHES (Version 1.1, Date: 29 /06/2020)

Project Title: Building an Evidence Base for Women-Specific Training and Injury Identification in Rugby Union – Global Questionnaire

Thank you for your time in taking part in the women's rugby union survey for 2020. The objective of this survey is to help to bridge the substantial gender data gap which exists in medical and sport science, particularly in contact sports. You are free to contact the research team at any time for any questions or ideas (details below):

Contact Details:

Dr Elisabeth Williams, ASTEM, College of Engineering, Bay Campus, Swansea University, Fabian Way, Swansea, SA18EN, United Kingdom: e.m.p.williams@swansea.ac.uk

Alternative Contacts: Dr Anna Stodter - anna.stodter@anglia.ac.uk; Dr Izzy Moore - imoore@cardiffmet.ac.uk; Dr Genevieve Williams - G.K.R.Williams@exeter.ac.uk

Invitation Paragraph

My name is Dr Elisabeth Williams and I am a senior lecturer in applied biomechanics at Swansea University in Wales, U.K. On behalf of the research team, we would like to thank you for your time and interest in completing this questionnaire.

What is the purpose of the study?

The aim of this survey questionnaire is to gather data about the sport of women's rugby

players at all levels; local club, university, elite, professional and international. You will be aware that professionalisation of women's rugby has come 33 years after the male game. Currently, worldwide women's participation in rugby is rising by 28% per year, with 2.7 million registered players at the end of 2018. As rugby has historically been a male-dominated sport, most research about training techniques and injury risk has focussed on male athletes.

The following has motivated us to carry out this project:

- Male-based findings in sports and medicine are routinely generalised to women, even when there is little to no evidence about how women respond. This has created a significant gender data gap
- What this ultimately means is 'women are not small men' and we need to ensure we are training women appropriately to make them better, safer athletes. This very much applies to sports related mild traumatic brain injury (mTBI), which is a serious problem in contact sports like rugby
- There are many physical differences between males and females, in addition to playing opportunities and access to expert coaching, which may compromise the safety and performance of female rugby players
- Reports have shown women to be 2.6 times more likely to suffer a concussion than men.
- Research shows that female athletes take longer to return to play, have worse symptoms and are more likely to report dizziness, fatigue and difficulty concentrating than males
- Differences between male and female head/neck physiology, neck strength and neuronal structure in the brain have been linked to a greater head injury severity in females following rugby impacts

Why have I been chosen?

You have been approached via women's rugby social media platforms to participate in this study as you are involved in coaching women's rugby players and you are over the age of 18. We would like to know about your experiences, the anthropometrics of different playing positions, injury patterns and health monitoring practices in particular. The data you provide will help us to create an evidence base to develop targeted, women-specific interventions to improve player safety. We hope to have as many women rugby players and coaches as possible complete this survey in order to build a valuable and informative dataset that we can all benefit from.

Taking part in this study is entirely voluntary. The questionnaire responses are completely anonymous, even to the research team. **As your responses for each**

question are automatically saved when you answer them, and no identifiable information is recorded in this study, we are unable to withdraw your data once you have provided a response.

What will happen to me if I take part?

Participation in this study involves completing the anonymous questionnaire which follows this document, after you click to confirm that you are over 18 and fit the criteria for the study. The questionnaire is in three sections;

- **Section One:** Demographics, playing experience and positions, playing level and basic health information
- **Section Two:** Concussion history, strength and conditioning, injury prevention training and injury management.
- **Section Three:** Female-specific concussion symptoms, female physiology including awareness of the effects of menstrual cycle on training, performance and injury risk. Questions regarding athlete medical support and financial resources in women's rugby union. This section will also ask you about possible support for a women-specific training curriculum pre-contact for players new to the sport.

The questionnaire should take you approximately ten minutes for section 1 and depending on the length of your answers, 15-20 minutes each for sections 2 and 3.

What are the possible disadvantages of taking part?

Some questions will ask you about previous concussions or possible concussions you may have witnessed. This may trigger upsetting memories, depending on the circumstances surrounding your injuries. If you do feel upset by any content in the questionnaire, you are advised to contact someone you trust and you are always welcome to contact anyone from the research team, whose email addresses are provided above.

What are the possible benefits of taking part?

By taking part in this study, you will be making an invaluable contribution to the much needed knowledge about women's rugby union. This information will be used to improve training strategies to minimise the risk of brain injury to women's rugby players and other contact sport athletes. The data gathered from this questionnaire will also help us understand what most women's rugby players and coaches know about how women's bodies respond to trauma. It will also help us gauge the extent to which players and coaches understand and use the menstrual cycle as a training aid. With that information we can work with rugby organisations to improve training practices and guidelines

specific to female athletes. In addition to publishing our generalised findings in open-source journals, we will be making our generalised findings available on Twitter: @TBI_ResearchNet.

Will my taking part in the study be kept confidential?

Due to the nature of this anonymous survey, your identity will not be recorded. The anonymised data will be kept by the research team and used both directly in publishable studies and as pilot information for three years. After this date, the data will be stored for a further four years for reference for future work in this area. All anonymised data will be kept on a secure cloud platform, accessible only by the research team.

Here is a link to a news article from early 2020 to provide you with more context for this study:

https://www-bbc-co-uk.cdn.ampproject.org/c/s/www.bbc.co.uk/news/amp/uk-wales-51434749

Data Protection and Confidentiality: Your data will be processed in accordance with the Data Protection Act 2018 and the General Data Protection Regulation 2016 (GDPR). All information collected about you will be kept strictly confidential. Your data will only be viewed by the researcher/research team. All electronic data will be stored on a password-protected computer file at Swansea University. Your consent information does not require your name to eliminate risk in the event of a data breach. Please note that as data is being collected online, once the data has been submitted online you will be unable to withdraw your information. **Data Protection Privacy Notice:** The data controller for this project will be Swansea University. The University Data Protection Officer provides oversight of university activities involving the processing of personal data, and can be contacted at the Vice-Chancellors Office. Your personal data will be processed for the purposes outlined in this information sheet. Standard ethical procedures will involve you providing your consent to participate in this study by completing the consent form that has been provided to you. The legal basis that we will rely on to process your personal data will be necessary for the performance of a task carried out in the public interest. This public interest justification is approved by the College of Engineering Research Ethics Committee, Swansea University. The legal basis that we will rely on to process special categories of data will be processing is necessary for archiving purposes in the public interest, scientific or historical research purposes or statistical purposes. **How long will your information be held?** We will hold any personal data and special categories of data for seven years. **What are your rights?** Please visit the University Data Protection webpages for further information in relation to your rights. Any requests or objections should be made in writing to the University Data Protection Officer: University Compliance Officer (FOI/DP), Vice-Chancellor's Office, Swansea University, Singleton Park, Swansea, SA2 8PP. Email: dataprotection@swansea.ac.uk **How to make a complaint:** If you are unhappy with the way in which your personal data has been processed, you may in the first instance contact the University Data Protection Officer using the contact details above. If you remain dissatisfied, then you have the right to apply directly to the Information Commissioner for a decision. The Information Commissioner can be contacted at: Information Commissioner's Office, Wycliffe House, Water Lane, Wilmslow, Cheshire, SK9 5AF, www.ico.org.uk 8**. What if I have any questions?** Re-iterate that further information can be obtained from the researcher contact stated above. Also state that the project has been approved by the College of Engineering Research Ethics Committee at Swansea University. If you have

any questions regarding this, any complaint, or concerns about the ethics of this research please contact Dr Andrew Bloodworth, Chair of the College of Engineering Research Ethics Committee, Swansea University. A.J.Bloodworth@swansea.ac.uk. The institutional contact for reporting cases of research conduct is Registrar & Chief Operating Officer Mr Andrew Rhodes. Email: researchmisconduct@swansea.ac.uk. Further details are available at the Swansea University webpages.

1. I confirm that I have read and understood the information sheet dated 29/06/2020, version number 1.1 and that I have the opportunity to contact members of the research team to ask questions. I understand that my participation is voluntary, and the questionnaire responses are completely anonymous, even to the research team. I understand that my responses for each question are automatically saved when I answer them, and no identifiable information is recorded in this study. I understand that I am unable to withdraw my data once I have provided a response. I understand that data obtained may be looked at by responsible individuals from Swansea University or from regulatory authorities where it is relevant to my taking part in research. I give permission for these individuals to have access to these records. I understand that data I provide may be used in reports and academic publications in anonymous fashion.

- ○ I agree to take part in this study

Page 2: Section One: Demographics, player and coach experience, different playing positions, playing level and collection and understanding of basic health information.

2. What country are you based in?

2.a. If you selected Other, please specify:

3. Optional: Please state the region where you currently coach:

4. Which of the following best describes your current coaching role?

4.a. If you selected Other, please specify:

5. Please select your age

[ ]

6. Have you ever played competitive rugby?

- Yes
- No

6.a. Do you currently play competitive rugby in addition to coaching?

- Yes
- No

6.b. Please select what best describes your current or former playing level (select all that apply)

- [ ] Recreational
- [ ] University
- [ ] Club second division
- [ ] Club first division
- [ ] Premier club
- [ ] International
- [ ] Other

6.b.i. If you selected Other, please specify:

[ ]

7. How many years have you been coaching rugby for?

- Less than 1
- 1
- 2
- 3
- 4
- 5
- 6
- 7
- 8
- 9
- 10+

8. Which of the following best describes your current level of coaching qualification?

- No formal qualification
- Entry level (e.g. Rugby Ready / Scrum Factory)
- Level 1
- Level 2
- Level 3
- Level 4
- Other

8.a. If you selected Other, please specify:

9. What is the highest level of women's rugby you have coached in the last 12 months?

- ○ Youth club
- ○ Adult club
- ○ International age group
- ○ Adult professional
- ○ International
- ○ University team
- ○ Other

9.a. If you selected Other, please specify:

10. What age group(s) of women's rugby players do you currently coach? (select all that apply)

- ☐ Under 7
- ☐ Under 10
- ☐ Under 15
- ☐ Under 16
- ☐ Under 18
- ☐ Social club
- ☐ University 1st team
- ☐ University 2nd/3rd team
- ☐ Senior club
- ☐ Premier club
- ☐ Professional
- ☐ National team/international

- [ ] Other

10.a. If you selected Other, please specify:

11. How many sessions per week do you coach women's rugby union (if this includes multiple teams please click other and provide the number and level of teams)

- [ ] Casual hours (less than one session per week)
- [ ] 1 session per week
- [ ] 2 sessions per week
- [ ] 3 sessions per week
- [ ] 4 sessions per week
- [ ] 5 sessions per week
- [ ] More than 5 sessions per week
- [ ] Other

11.a. If you selected Other, please specify:

12. Does the team(s) you coach have access to other providers? (i.e. physiotherapists, strength and conditioning coach). Please select all that apply:

- [ ] Physiotherapist
- [ ] Strength and conditioning coach
- [ ] Medical team
- [ ] Fitness coach
- [ ] Other

12.a. If you selected Other, please specify:

13. Do you coach any other sports in addition to rugby?

- ( ) Yes
- ( ) No

13.a. Please select the other sport(s) you coach

- [ ] Football
- [ ] Netball
- [ ] Swimming
- [ ] Athletics
- [ ] Field Hockey
- [ ] Martial Arts (please state)
- [ ] Other

13.a.i. If you selected Other, please specify:

14. Are you familiar with the term concussion?

- ○ Yes
- ○ No

15. Please select all statements you think are true/correct

- ☐ Concussion can be caused by an impact to the head
- ☐ Concussion can be caused by an impact to the body
- ☐ Concussion only happens when a person is knocked out/loses consciousness
- ☐ Concussion only happens in rugby/contact sport
- ☐ Concussion can be caused by a direct or indirect blow to the head
- ☐ Most concussions don’t involve loss of consciousness
- ☐ All concussions get reported and documented
- ☐ Most concussions go unreported and undiagnosed

16. Which of the following are common signs of concussion? (select all that apply)

- ☐ Arm pain
- ☐ Chest pain
- ☐ Confusion
- ☐ Cut to the face
- ☐ Difficulty concentrating

- [ ] Dizziness
- [ ] Drowsiness
- [ ] Feeling or being sick
- [ ] Headache
- [ ] Knocked out
- [ ] Memory loss
- [ ] Neck pain
- [ ] Nosebleed
- [ ] Stomach cramps

17. How many times have you seen a player in your team concussed?

- ○ None
- ○ 1-10
- ○ 11-20
- ○ 21-40
- ○ 40 +

18. Who is in charge of your players’ return to play following injuries, including concussion?

- ○ Team doctor
- ○ Team physiotherapist
- ○ Players own doctor (separate to team)
- ○ Other

18.a. If you selected Other, please specify:

19. During a match or training, if you thought a player was concussed what do you think you **should** do?

- ☐ Remove them immediately
- ☐ Check on them in 5 minutes to see if they have progressed
- ☐ Give them a head guard
- ☐ Consult with the medical team (if present)
- ☐ Nothing
- ☐ Other

19.a. If you selected Other, please specify:

20. During a match or training, if you thought a player was concussed what **would** you do?

- ☐ Remove them immediately
- ☐ Check on them in 5 minutes to see if they have progressed
- ☐ Give them a head guard
- ☐ Consult with the medical team (if present)
- ☐ Nothing
- ☐ Other

20.a. If you selected Other, please specify:

21. During a match, if a player showed signs of concussion but insisted they were fine, what do you think you **should** do?

- ☐ Allow them to play on, it’s the players decision
- ☐ Try to persuade them to come off but if they refuse allow them to continue
- ☐ Allow them to play for another 5 minutes to see if they have progressed
- ☐ Consult the referee
- ☐ Insist they leave the pitch
- ☐ Consult with the medical team (if present)
- ☐ Other

21.a. If you selected Other, please specify:

22. During a match, if a player showed signs of concussion but insisted they were fine, what **would** you do?

- ☐ Allow them to play on, it’s the players decision
- ☐ Try to persuade them to come off but if they refuse allow them to continue
- ☐ Allow them to play for another 5 minutes to see if they have progressed

- [ ] Consult the referee
- [ ] Insist they leave the pitch
- [ ] Consult with the medical team (if present)
- [ ] Other

22.a. If you selected Other, please specify:

23. Have you noticed your players feeling excessively tired, irritable, confused, had concentration problems or other concussion symptoms following a match but not told you?

- ( ) Yes
- ( ) No
- ( ) Other

23.a. If you selected Other, please specify:

24. If you have previously been informed that a player may be experiencing concussion symptoms, did you speak to that player(s)?

- ( ) Yes
- ( ) No

- Other

24.a. If you selected Other, please specify:

24.b. How did the player respond?

- Brushed off their symptoms, seem eager to continue playing/training
- Wanted me to decide on their return to play
- Wanted me to remove them from play
- Helped me to understand what they were feeling and then they suggested medical advice
- They were actively seeking medical advice
- They didn’t wish to speak with me about it
- Other

24.b.i. If you selected Other, please specify:

25. Have you ever seen other coaching staff allowing a player to continue, when you thought they were concussed?

- Yes
- No
- Prefer not to say

- Other

25.a. If you selected Other, please specify:

26. Following a rugby injury you have seen (excluding possible concussion), did you feel there was adequate pitch-side medical provision in terms of injury **identification:**

- Yes
- No
- Other

26.a. If you selected Other, please specify:

27. Following a rugby injury you have seen (excluding possible concussion), did you feel there was adequate pitch-side medical provision in terms of injury **management?**

- Yes
- No
- Other

27.a. If you selected Other, please specify:

28. Following a rugby injury you have seen (excluding possible concussion), did you feel there was adequate pitch-side medical provision in terms of injury **rehabilitation?**

- Yes
- No
- Other

28.a. If you selected Other, please specify:

29. Following a previous rugby **head injury or concussive injury** (in your team/opposition), did you feel there was adequate medical provision in terms of injury **identification:**

- Yes
- No
- Other

29.a. If you selected Other, please specify:

30. Following a previous rugby **head injury or concussive injury** (in your team/opposition), did you feel there was adequate medical provision in terms of injury **management:**

- Yes
- No
- Other

30.a. If you selected Other, please specify:

31. Following a previous rugby **head injury or concussive injury** (in your team), did you feel there was adequate medical provision in terms of injury **rehabilitation:**

- Yes
- No
- Other

31.a. If you selected Other, please specify:

32. If a player you coach has suffered a previous concussion, how long do you think it will take from them to fully recover (feeling as well as they did before the injury, with no concussion symptoms present)?

- Within 1 week
- Within 2 weeks
- Within 1 month
- Up to 3 months
- Beyond 3 months
- Other

32.a. If you selected Other, please specify:

33. When someone you coach has returned after a bodily injury (excluding head injury or concussion), are you confident that they are fit enough to play:

- Yes- I ensure they have fully recovered
- Yes- I followed a return-to-play protocol guided by a medical team
- No- I don’t feel they are fully recovered but it is down to the player when they return to play
- Other

33.a. If you selected Other, please specify:

34. When someone you coach returns to play after a **head injury or concussion,** are you confident that they are fit enough to play:

- Yes- I ensure they have fully recovered
- Yes- I followed a return-to-play protocol guided by a medical team
- No- I don’t feel they are fully recovered but it is down to the player when they return to play
- Other

34.a. If you selected Other, please specify:

35. If you have seen a player you coach experience a possible concussion, have you seen other players pressure them to return to play?

- Yes
- No
- Other

35.a. If you selected Other, please specify:

36. If you have seen a player you coach experience a possible concussion, have you or other coaches/officials pressured them to return to play?

- Yes
- No
- Other

36.a. If you selected Other, please specify:

36.b. Please explain your reasoning for pressuring the player to return to the game. (Optional)

37. If you have seen an opposition player experience a possible concussion during a match, have you witnessed their coaches/officials pressuring them to return to play?

- Yes
- No
- Other

37.a. If you selected Other, please specify:

38. If you have seen an opposition player experience a possible concussion during a match, have you witnessed their teammates pressuring them to return to play?

- Yes
- No
- Other

38.a. If you selected Other, please specify:

38.b. Please explain what you witnessed (Optional)

39. During your coaching career, have you ever taken part in any educational sessions and/or medic workshops about sporting injuries?

- Yes

○ No

39.a. Please state what these workshops were called and if possible, what organisations these were organised by (optional).

39.b. Have any of these sessions included concussion education specifically?

○ Yes
○ No
○ Other

39.b.i. If you selected Other, please specify:

40. During your coaching career have you ever thought about the role of neck strength for injury prevention and/or performance?

○ Yes
○ No
○ Other

40.a. If you selected Other, please specify:

40.b. Have you ever considered the role of neck strength in minimising the risk of concussion injuries?

- Yes
- No
- Other

40.b.i. If you selected Other, please specify:

Page 3: Section Two: Strength and conditioning practices and experience, concussion knowledge and experience, injury prevention training and management strategies.

41. For the women's rugby team(s) you coach, in a normal year, how long is your pre-season training block?? (from the time the players meet as a group)

41.a. If you selected Other, please specify:

42. For the women's rugby team(s) you coach, what does your pre-season typically include? (select all that apply)

- ☐ Running endurance and speed
- ☐ Strength and gym work
- ☐ Ball/passing skills
- ☐ Team tactics
- ☐ Contact drills and/or tackle technique
- ☐ Falling technique and/or body awareness training
- ☐ Active recovery sessions
- ☐ Other

42.a. If you selected Other, please specify:

43. Approximately how many hours of contact activities (e.g. using tackle shields, tackling, tackle technique, rucking etc.) do you deliver each week during the playing season? (please give an average value across the season)

- None
- Less than one hour per week
- One dedicated contact session per week
- Two dedicated contact sessions per week
- Three dedicated contact sessions per week
- Four + dedicated contact sessions per week
- Other

43.a. If you selected Other, please specify:

44. Approximately how many hours of non-contact training (team fitness, passing, skills, agility drills, touch rugby) do you deliver each week during the playing season? (please give an average value across the season)

- None
- Less than one hour during the week
- One dedicated non-contact session per week
- Two dedicated non-contact sessions per week

- Three dedicated non-contact sessions per week
- Four + dedicated non-contact sessions per week
- Other

44.a. If you selected Other, please specify:

45. Approximately how many hours of strength training (weights, plyometrics etc.) do you prescribe for your players each week during the playing season?

- None
- Less than one hour during the week
- One dedicated non-contact session per week
- Two dedicated non-contact sessions per week
- Three dedicated non-contact sessions per week
- Four + dedicated non-contact sessions per week
- Other

45.a. If you selected Other, please specify:

46. Approximately how many hours of endurance training (running, long intervals, etc.) do you prescribe for your players each week during the playing season?

- None
- Less than one hour during the week
- One dedicated non-contact session per week
- Two dedicated non-contact sessions per week
- Three dedicated non-contact sessions per week
- Four + dedicated non-contact sessions per week
- Other

46.a. If you selected Other, please specify:

47. Approximately how many hours of speed training (sprinting, short intervals etc.) do you prescribe for your players each week during the playing season?

- None
- Less than one hour during the week
- One dedicated non-contact session per week
- Two dedicated non-contact sessions per week
- Three dedicated non-contact sessions per week
- Four + dedicated non-contact sessions per week
- Other

47.a. If you selected Other, please specify:

48. Are you typically able to deliver the amount of coaching time for contact activities as you intended?

- Yes
- No
- Other

48.a. If you selected Other, please specify:

49. How confident do you feel in coaching contact/tackle technique and contact intensity?

- Not confident at all (0/5)
- A little confident (1/5)
- Somewhat confident (2/5)
- Relatively confident (3/5)
- Comfortably confident (4/5)
- Very confident (5/5)
- Other

49.a. If you selected Other, please specify:

50. When completing formal coaching qualifications, did you complete training to teach players how they should fall or land on the ground?

- Yes
- No
- Other

50.a. If you selected Other, please specify:

50.b. How was this taught to you? I.e. videos, written work, face to face learning?

50.c. What were the fall techniques recommended (parachute, land on front, side etc), if you are unsure of the terms, please give a description:

50.d. Have you used falling technique training in your coaching practice?

- No
- Yes, once
- Yes, once every season
- Yes, once every month
- Yes, weekly

○ Other

50.d.i. If you selected Other, please specify:

50.d.ii. Why do you not use falling technique training in your coaching practice?

50.d.iii. What is your rationale for teaching falling technique training in your coaching practice?

51. How would you like to receive falling training if it were to be provided to you? (e.g. webinar series, visiting coaching specialist, online courses with feedback provided).

52. Have you ever encouraged your players to do extra training that focused on improving neck strength?

- No and I’m not interested in doing it
- No, but it’s something I would be keen to try in the future
- Yes, when I coach I encourage everyone to do basic neck strength exercises
- Yes, either myself, the strength and conditioning coach or fitness coach regularly prescribe/coach bodyweight neck strengthening exercises
- Yes, either myself either the coach,, the strength and conditioning coach or fitness coach regularly prescribe/coach weighted neck strengthening exercises
- Other

52.a. If you selected Other, please specify:

53. In your opinion, what do you think is the most common mechanism of head impact, resulting in potential concussive injuries in women’s rugby at the level you coach at? Examples can influence direct contact with another player’s head, direct contact between the head and the ground, direct contact as a tackler, as a ball carrier, head to knee, or foot of another player etc.

Page 4: Section Three: Experience, knowledge, understanding and perceptions of injury prevalence and performance specifically in relation to the menstrual cycle.

The following questions are based around the menstrual cycle and performance. Please answer as a coach.

54. Regarding the effects the menstrual effect has on players’ performance and training, are or other coaching/medical team staff (i.e. physio, S&C coach) aware of players' menstrual cycle?

- We track all player’s cycles and relate this to our training program
- We track all player’s cycles but don't relate it to training
- We don’t track any player’s cycles or ever talk about this
- Other

54.a. If you selected Other, please specify:

55. Regarding the effects the menstrual effect has on players’ physical performance, if a player is visibly struggling with training or tells you they are, do you ask them what stage of their menstrual cycle they are at?

- Yes
- No
- Other

55.a. If you selected Other, please specify:

56. Please select all of the following which apply to you as a coach and your team:

- ☐ We use a menstrual cycle tracking app which all the players update on their devices and share with the coaching/medical staff
- ☐ We are aware of which players take hormonal contraception
- ☐ We track all player's cycles and tailor the training program accordingly
- ☐ We track some of our player's cycles and where possible, tailor the training program accordingly
- ☐ The issue of menstrual cycle is regularly discussed among coaches and players in our team
- ☐ I am aware how dietary demands change during the menstrual cycle and advise players accordingly
- ☐ I don't know at what stage of the menstrual cycle performance might be impaired or enhanced
- ☐ We have never talked about this and I think players should train hard regardless
- ☐ I don't know how to have conversations with players about their menstrual cycles
- ☐ Other

56.a. If you selected Other, please specify:

57. Do the players you coach receive pre-season health and/or medical screening,cognitive testing/screening or baseline concussion testing e.g. SCAT5?

- ○ Yes
- ○ No
- ○ Other

57.a. If you selected Other, please specify:

57.b. What does this testing include? (select all which apply)

- ☐ Baseline concussion testing (e.g. SCAT5, Cogsport, please specify below)
- ☐ Menstrual cycle/hormonal contraceptive history
- ☐ Fitness testing
- ☐ Injury history
- ☐ Other

57.b.i. If you selected Other, please specify:

57.c. If your players do undergo preseason fitness and cognitive testing, did you ask players at what stage they are at in their menstrual cycle, or are you aware of this via an menstrual tracking app?

- ○ Yes this included natural cycle or taking hormonal contraceptives
- ○ Yes this include the natural cycle including what day they were on

- No
- Other

57.c.i. If you selected Other, please specify:

58. If players have previously experienced concussion, did you or other medical members ask / were you aware of what stage of their menstrual cycle they were at when this occurred?

- Yes
- No
- Other

58.a. If you selected Other, please specify:

58.b. Why did you enquire about the stage of the player’s menstrual cycle when they suffered a concussion? (please select all that apply)

- ☐ I understand that menstrual cycle can affect progesterone levels which, when high, may have a protective effect on the player’s brain
- ☐ I understand that the menstrual cycle can have significant effects on all aspects of a player’s physical and cognitive performance
- ☐ I believe it is important to collect data to determine whether my player’s concussive symptoms are worse during different phases of their cycle

- [ ] I was told that this is important to know but I do not understand why
- [ ] Other

58.b.i. If you selected Other, please specify:

59. In your opinion do you feel that performance is altered when athletes train/play during certain times of their menstrual cycle?

- ○ Yes
- ○ No
- ○ I don't know
- ○ Other

59.a. If you selected Other, please specify:

60. Do you have any further comments to make about women’s rugby union training and coaching, which you believe are important for future research?

Page 5: Thank you!

The research team would like to sincerely thank you for your time and participation in this world wide women's rugby union survey.

We believe that women athletes should be trained like women athletes, and not like they are just small men.

Please share with all the women rugby coaches over the age of 18 you know, from anywhere, at any level!

Keep an eye out for our findings over the next six months by following @TBI_ResearchNet Twitter.

Key for selection options

2 - What country are you based in?

Algeria
American Samoa
Andorra
Antigua and Barbuda
Argentina
Aruba
Australia
Austria
Azerbaijan
Bahamas
Barbados
Belarus
Belgium
Bermuda
Bosnia and Herzegovina
Botswana
Brazil
British Virgin Islands
Brunei
Bulgaria
Burundi

Cameroon
Canada
Cayman Island
Chile
China
Chinese Taipei
Colombia
Cook Islands
Costa Rica
Croatia
Cuba
Curaçao
Cyprus
Czech Republic
Denmark
Dominican Republic
Ecuador
El Salvador
England
Estonia
Eswatini
Fiji
Finland
France
Georgia
Germany
Ghana
Greece
Guadeloupe
Guam
Guatemala
Guyana
Hong Kong
Hungary
Iceland
India
Indonesia
Iran
Ireland (Republic of)
Israel

Italy
Ivory Coast
Jamaica
Japan
Kazakhstan
Lao
Latvia
Liechtenstein
Lithuania
Luxembourg
Madagascar
Malaysia
Mali
Malta
Martinique
Mauritius
Mexico
Moldova
Monaco
Mongolia
Montenegro
Morocco
Namibia
Netherlands
New Zealand
Nigeria
Niue
Northern Ireland
Norway
Pakistan
Panama
Papua New Guinea
Paraguay
Peru
Phillipines
Poland
Portugal
Romania
Russia
Rwanda

Samoa
San Marino
Scotland
Sengal
Serbia
Singapore
Slovakia
Slovenia
Solomon Islands
South Africa
South
Spain
Sri Lanka
St. Kitts and Nevis
St. Lucia
St. Vincent & The Grenadines
Sweden
Switzerland
Tahiti
Tanzania
Thailand
Tonga
trinidad and Tobago
Turkey
Turks and Caicos Islands
Ukraine
United
USA
Uruguay
Uzbekistan
Vanuatu
Wales
Other

4 - Which of the following best describes your current coaching role?

Director of Rugby/Head coach
Assistant coach
Forwards coach
Backs coach
Kicking/skills coach

Other

5 - Please select your age

18
19
20
21
22
23
24
25
26
27
28
29
30
31
32
33
34
35
36
37
38
39
40
41
42
43
44
45
46
47
48
49
50
51
52
53

54
55
56
57
58
59
60+

41 - For the women’s rugby team(s) you coach, in a normal year, how long is your pre-season training block?? (from the time the players meet as a group)

Training is only completed within the season
One to two weeks (training less than 3 times per week)
Three to six weeks (training less than 3 times per week)
One to two weeks (training 3+ times per week)
Three to four weeks
Five to six weeks
Other
